# Affective health and countermeasures in long-duration space exploration

**DOI:** 10.1016/j.heliyon.2022.e09414

**Published:** 2022-05-13

**Authors:** Matteo Gatti, Rocco Palumbo, Alberto Di Domenico, Nicola Mammarella

**Affiliations:** Department of Psychological Sciences, Health and Territory, University of Chieti, Italy

**Keywords:** Emotion, Affective health, Resilience, Mood, Stress, Space mission

## Abstract

**Background:**

Space research is shifting attention toward interplanetary expeditions. Therefore, whether long-duration spaceflight may influence affective health is becoming an urgent issue.

**Method:**

To this end, we undertook a literature search and reviewed several behavioral simulation studies on Earth that focused on affective components in space. We concluded with studies showing how spaceflight can impact on affective health of astronauts with a positively laden trajectory.

**Results:**

By analyzing the multifaceted theoretical concept of affective health, we show that there is a variety of affective states (e.g., stress, coping, adaptation, and resilience) that can be differently affected by spaceflight.

**Conclusions:**

Countermeasures geared toward promoting positive emotions could play a key role in positive adaptation to extreme environments and thus during long-duration space missions may benefit. Subjective resilience plays a mediating role in adaptation, but its definition needs to be deepened in order to develop robust countermeasures that may prevent the onset of emotional disorders.

## 1 Introduction

Many space agencies are shifting their resources and attention towards Mars ‘colonization’. As of February 2021, when the Perseverance probe landed on the soil of the red planet and sent back the first images of what could be our second home, interplanetary missions are perceived as more feasible.

Researchers agree that spaceflights out of Orbit will give rise to different needs compared with those observed in Orbit (Gushin et al., 2021). In fact, an orbital spaceflight is a source of psychological and interpersonal distress for crewmembers, but the amount of effort required to adapt to a Long-Duration Space Mission (LDSM) may indeed increase dramatically. During an interplanetary mission, cosmonauts can experience a strong sense of isolation and separation from Earth due to communication delays (one-way communication delay is over 20 min), to infrequent refuelling, and to the “Earth out of sight” phenomenon (Kanas and Manzey, 2008). These factors can affect astronauts' bodies and minds, as well as produce feelings of dullness and nostalgia and put their Affective Health (AH) at risk (Kanas, 2015). In fact, we know that negative emotions can negatively affect cognition and performance (Fredrickson, 2004, 2008) and exert dangerous effects on human performance in Space. Therefore, AH is a pressing issue in Long-Duration Space Exploration (LDSE).

The goal of this review is threefold. First, to better define the concept of AH (often used as an umbrella term) within the space sciences research by adopting the Gross's (2015) model as a theoretical guide. Second, to describe the behavioral studies performed in terrestrial Space analogs on emotions and emotion-related components (mood, stress, coping) that may allow a higher level of adaptation to extreme environments. Third, to describe the countermeasures that have been developed to decrease the emotional impact of a spaceflight and that can be viewed as the most appropriate for LDSE.

## 2 Methods

This review was conducted and recoded according to the PRISMA (Preferred Reporting Items for Systematic Reviews and MetaAnalyses) Statement (Liberati et al., 2009).

### 2.1 Search strategy and selection criteria

The present review on the emotional responses, health and countermeasures in space exploration was conducted via computer searches of the PubMed, PsycINFO, Scopus, and Google Scholar databases. The searches were performed until the 20^th^ of January, 2022, using the keywords “emotional health”, “adaptation”, “affect”, “stress”, “mood”, “emotion”, “emotion responses”, “space”, “spaceflight”, “long duration space exploration”, “head down bed rest”, “space analogs”, “terrestrial simulation of space environment”, “extreme environments”, and “countermeasures”.

In addition, we searched for some combinations of previous keywords: “emotion responses in long-duration missions”, “emotion responses in long-duration Space exploration”, “adaptation extreme environments”, “countermeasures extreme environment”, “countermeasures long-duration missions”.

### 2.2 Inclusion/exclusion criteria

We included studies with the following criteria:

1) Works providing a definition of affective and emotional health, and/or highlighting the relationship between health and affects (emotions, moods, stress responses, coping strategies).
2) Works focusing on the relationships between affects and interpersonal factors (e.g. communications, cultural/gender/personality differences, humor, etc.) in the adaptation to extreme environments, spaceflights, and space analogs; we included here surveys and astronauts' diaries and recounts.
3) Works on the emotional countermeasures adopted in space missions and/or those offering suggestions for countermeasures during interplanetary missions.
4) Written in English.

The exclusion criteria were considered as follows:

1) Unrelated topic.
2) Studies without sufficient data provided (e.g., study site, study period, and sample size).
3) Interventional studies/case reports/Editorial comments/Letters to Editor, etc.

### 2.3 Study selection

**Figure 1:**
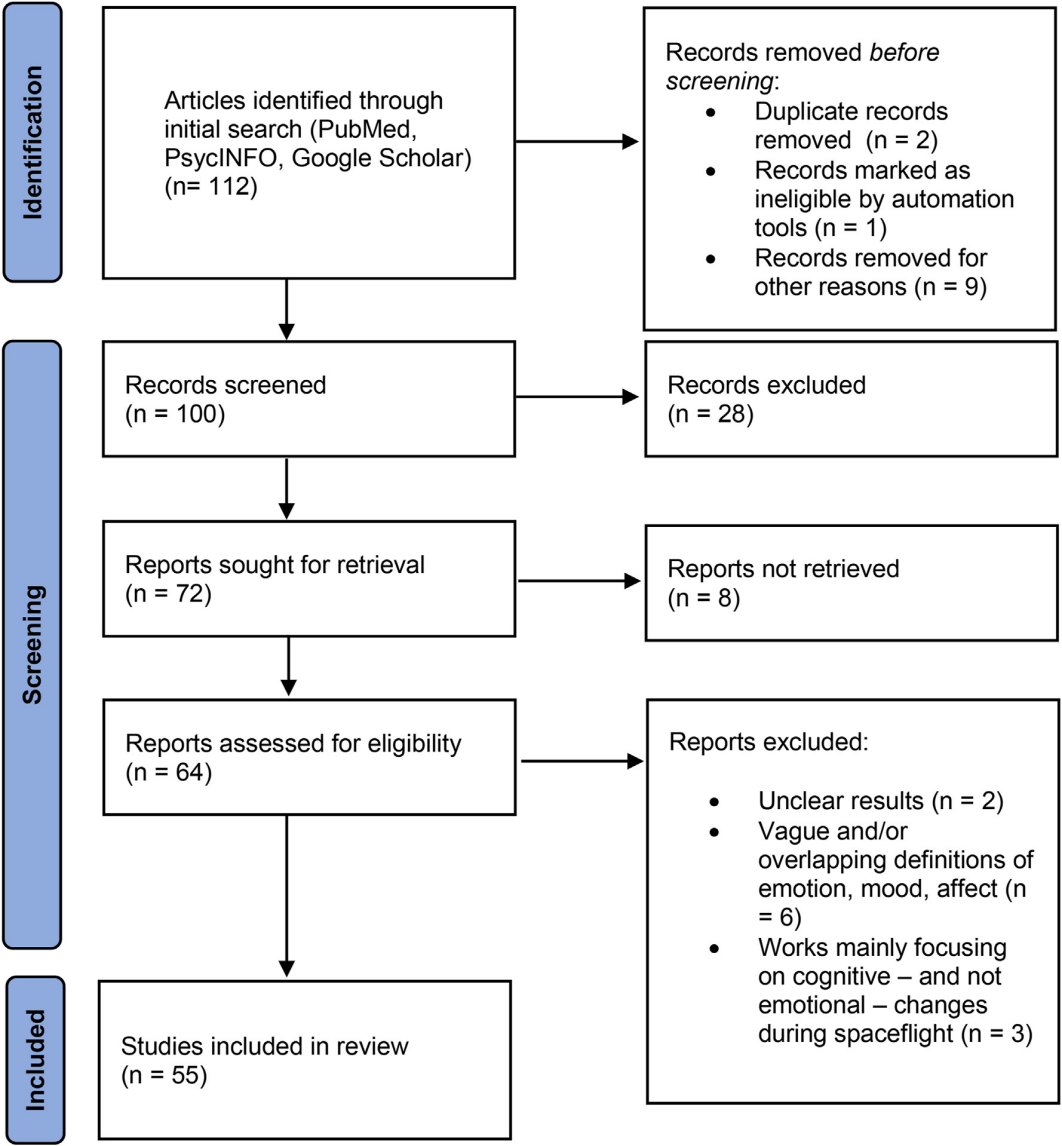
Flowchart describing the search strategy and inclusion/exclusion of studies by following the Preferred Reporting Items for Systematic Reviews and MetaAnalyses (PRISMA, 2009) guidelines.

The study selection processes are depicted in Figure 1. Initially, we identified the articles according to the PRISMA guidelines and listed them in an excel table. In the next step, titles and abstracts that were irrelevant to the topic or duplicate records were removed. Therefore, in the eligibility check phase, we went through the full text. In the final stage, all items selected in the previous stages have been reviewed or checked for quality assessment; low-quality studies were excluded.

### 2.4 Some basic concepts about emotions

The definition of emotions has a long research tradition in psychology and different explanations have been put forward to account for emotion-based behavioral responses (e.g., stress). For instance, emotions have been viewed as being categorical (e.g., Ekman, 1994) and dimensional (e.g., Posner et al., 2005) or with a different level of intensity (e.g., the joy can be either joy, serenity or euphoria, e.g., Plutchik, 1980). In addition, emotions are typically classified in “primary” and “secondary”: primary emotions are considered innate (e.g., fear, joy, sadness, etc.), whereas secondary emotions (e.g., shame, guilt, etc.) are more complex and require the involvement of some basic cognitive processes (Damasio, 1994; Fragopanagos and Taylor, 2005).

The terms emotion and mood are often used interchangeably because they can be referred to a positive or negative connotation, but they would primarily involve a different object and a different duration. Emotions develop over a short time and are triggered by a specific object or event (e.g., being overjoyed on your wedding day); moods, on the other hand, are diffuse, pervasive, and have no manifest objects (e.g., feeling sad or melancholy). Among the many emotion-based responses, stress is the most studied and refers to a negative emotional state resulting from inability to cope with a situation (Gross, 2015). In particular, individuals experience a complex array of negative emotions in front of a challenge or a particular demand. During the recent COVID-19 pandemic, for instance, the effect of fearful emotions has been explored on personal expectations about future (Ceccato et al., 2021), as well as on buying/accumulation behaviors (Cannito et al., 2021). Finally, research on emotions and stress-related responses is of great interest in the fields of machine learning, robotics, and artificial intelligence (Cowie et al., 2001; Fragopanagos and Taylor, 2005). As we will state in the section about countermeasures, the emotion-technology pair is of great interest for adaptation during LDSE missions.

Depending on the research objectives, emotions have been investigated with different approaches, but one of the most widely used in the cognitive domain, is the affective image content analysis (Zhao et al., 2021). This method investigates the relationships between visual stimuli and emotions in the context of a stressful situation or by referring to individual differences in stress responses. Many image databases have been developed to this aim, such as the International Affective Picture System (IAPS, see Lang et al., 1997), and more recently, a database of videotaped emotional stimuli, called the *Chieti Affective Action Videos* database (Di Crosta et al., 2020; La Malva et al., 2021).

In sum, emotions can be simple or complex, normal or pathological, positive or negative, and influenced by both external and internal factors (Kehoe et al., 2012; Mehrabian, 1996; Saraiva et al., 2013). In the present work, we will consider emotions, moods, and stress responses as instances of the category "affects" or "affective states" as described better in the next sections.

### 2.5 Emotional or affective health?

Long-Duration Space Explorations (LSDEs), or Long-Distance Space Exploration Missions (LDSEMs, see Bell et al., 2019), such as missions to Mars (Salas et al., 2015), may last up to 30 months (Drake, 2009). Until now, in short-duration missions, space agencies have dealt with the impact of emotional problems via both preventive (selection of the 'right stuff') and protective measures (countermeasures). However, these solutions may not be adequate for LSDEs, where even the most experienced astronauts can show adaptation problems and emotional disturbances (Alfano et al., 2021). For example, cosmonaut Lebedev (1988) during his 211-day mission on the space station Salyut 7 developed some typical depressive symptoms (e.g., irritability, anhedonia, and sleep disturbances). In addition, affective states disorders, if not promptly recognized, in the medium/long-term could affect group dynamics (e.g., emotional contagion, see Van Kleef et al., 2017), communications among crewmembers (Caddy, 2018), sleep quality, cognitive and executive functions, and, consequently, impact on performing emergency tasks (Brownlow et al., 2020).

In recent years, researchers and space agencies are becoming more aware about the role of psychosocial variables in spaceflights (e.g., Alfano et al., 2017; Kanas and Manzey, 2008) and emotional health is becoming a compelling issue. Despite this increasing interest, to date, researchers are still debating about the terminology to be used when referring to astronauts' mental health. In fact, if we try to list a series of similarities and differences between the "mental health," "behavioral health," "emotional health," “affective health", “psychological well-being" and/or "emotional well-being" concepts, we can soon realize that this is not an easy task. More specifically, the literature is poor, and definitions are often vague. Here are some of the definitions used for emotional health: a positive attitude toward oneself and the world based on the ability to turn negative emotions into positive ones (Tarabakina, 2000); psychological functioning that leads to positive change, personal growth, and self-confidence (Svishnikova et al., 2020); a positive state of emotions, self-esteem, and resilience that leads to self-actualization, self-efficacy, and health-promoting behaviors (Courtwright et al., 2020). The lack of a robust theoretical framework in the emotional health literature stems from multiple factors. First, there is a terminological confusion within the health research field per sé (Feller et al., 2018), as well as a deeper lexical (and conceptual) confusion about emotion-related terms (e.g., emotion, mood, feelings, etc., which are used interchangeably; see Gross, 1998, 2015; Lischetzke and Eid, 2011; Lochner, 2016). Second, there is a variety of approaches and methodologies within the study of emotions and their related domains in the aerospace psychology research (e.g., Mauss and Robinson, 2009). Although this methodological eclecticism deepened our understanding of astronauts’ emotions, it made difficult to compare results across studies. Third, during a space mission, astronauts are exposed to multiple environmental factors or stressors that can differentially influence emotion responses and these effects are difficult to disentangle.

An interesting first attempt to develop an emotional health framework for space mission is the one by Alfano and colleagues (2017) who claimed that the study of normal and abnormal emotion-related responses in LSDEs requires a "guiding theoretical framework” (Alfano et al., 2017, p. 26). They defined behavioral health (BH) as a "broad and all-encompassing term" (p. 4), which includes behavior, cognition, performance, and emotion responses. Albeit not synonymous (BH is a broader concept than mental health), it is preferable to use BH instead of mental health to avoid stigma. Indeed, emotions have received little attention in the space sciences compared to behavior and/or cognition, and although there are few data in the literature, we know that emotional maladjustment is a risk to overall health during a space mission. Etymologically, emotional health should refer to the health of the emotions, i.e., the complex pattern of reactions by which one attempts to cope with a personally significant issue or event. More specifically, according to Alfano et al. (2017), emotional health is the part of human health that is sustained by healthy emotion regulation processes, e.g., increase and decrease an emotion (Gross, 2015). However, emotional health should involve the functioning of affective regulation processes as well (see Gross, 2015, Gross et al., 2019) such as stress, coping strategies, adaptation, and resilience. Consequently, the adoption of the concept of Affective Health (AH) seems more warranted in space science research. In this regard, Huber et al. (2011)'s definition of health is in line with this assumption when they refer to "the ability to adapt and self-manage" (p. 2), as well as the ability to regain that sense of coherence, the so-called "recovery of coherence". In conclusion, affective states and their regulation are crucial for "good mental health" (Fusar-Poli et al., 2020). However, in order to reduce the likelihood of emotional, medical, and psychiatric disorders in LSDEs, researchers must consider affective regulation in spatial analogs clarifying the targeted affective state better (De La Torre, 2014; Van Baarsen et al., 2009). Here we will adopt the concept of AH throughout the paper and thus we will refer to the degree to which one's health is dependent on the functioning of affective regulation processes (i.e., stress and coping strategies). To this aim, we will first describe the different analogical techniques that have been used to study the role of affective states. Subsequently, we will focus on reviewing the main findings about the different components of AH (e.g., stress, coping, mood, etc.) and how they are affected during a simulation of a space mission.

### 2.6 Analogs for the study of affective health

Emotions and emotion-related domains research typically uses a variety of methods and methodologies, including self-reports, experimental manipulations, and psychobiological measures, to cite only few (see Mauss and Robinson, 2009 for a review). In space science research, in addition to real microgravity conditions (e.g., aboard the ISS, namely the International Space Station), affective domains have been investigated through anecdotal reports and analogic research, i.e., behavioral studies on Earth aimed at mimicking some features of the spatial environment.

Anecdotal reports are written and/or oral retrospective narratives of astronauts' recorded material; typically, this type of information is analyzed qualitatively, such as with TCA (thematic content analysis, Smith, 1993) to identify elements of interest in the narratives, e.g., coping strategies (see Suedfeld et al., 2009).

Earth-based analogs of Space are, instead, locations and/or circumstances on Earth that share some characteristics with Space and/or spaceflights. Each analog simulates unique physiological and behavioral effects associated with spaceflight, ranging from a bed to polar research stations, to undersea labs. An Earth-based analogic scenario allows scientists to study the effects of space stressors on human body and mind in a more controlled setting.

Physiological analogs and isolation and confinement analogs are the two main categories among terrestrial analogs techniques. Head-down bed rest (HDBR), dry immersion, and parabolic flight are the most well-known from the former. These are primarily used to investigate the effects of microgravity on human body and mind. Because volunteers lie down on a bed tilted at a 6-degree angle for a period ranging from several hours to several weeks, HDBR allows researchers to study microgravity effects on brain and cardiovascular system (e.g., see Chen et al., 2016; Liu et al., 2012, 2015; Mammarella, 2020a, 2020b). In the dry immersion procedure (DI), a volunteer is immersed in a tank surrounded by a bladder filled with thermoneutral water, so he or she is suspended in the water but remains dry and experiences a constellation of bed-rest-like effects on the muscular, bone, and cardiovascular systems (Navasiolava et al., 2011). Finally, parabolic flights entail driving an aircraft to a certain altitude and then shutting down the engines to descend in free fall, reproducing short periods of microgravity (20–25 s) repeatable several times in a row. However, they are more expensive and less accessible (see Pletser, 2020). Microgravity analogs allow for the lack of axial loads, a shift of body fluids towards the brain, hypokinesia, and a restriction to a recumbent position (Belavy et al., 2011; Pavy-Le Traon et al., 2007; Tomilosvakya et al., 2019).

Although there is no perfect analog capable of reproducing an entire spaceflight, choosing that particular simulation is determined by the research goals (e.g., it is not necessary to send volunteers to Antarctica if we want to study the effects of confinement on emotions) according to the pros and cons of each technique. For instance, physiological analogs require a long recruitment phase as only volunteers with a specific physical and psychological profile can be admitted. Differently, in ICE (isolated, confined, and extreme) or ICC (controlled) environments it is not possible to reproduce microgravity effects. In addition, the ecological validity across these techniques may vary (e.g., Cromwell& Neigut, 2021). To summarize, we looked at some simulation techniques in space science research in order to better describe how the impact of a space mission over AH has been studied. We agree with Watenpaugh (2016) that there are qualitative differences between simulative and real-world microgravity research, but we believe that analogic research remains a valid tool for clarifying the different trajectories that affective states may take in LDSEs as shown in the next paragraphs.

### 2.7 Stress-related and coping responses

Stress is a subjective and physiological response to one or more internally or externally generated stressors (e.g., a state of displeasure and high arousal, see Kristensen et al., 1998). A stressor is "a stimulus that affects an organism in an arousing manner," while stress "refers to the changes in an organism that are caused by a given stressor" (Kanas, 1990, p. 457). Stress can be pathogenic when causing negative symptoms or salutogenic when leading an individual to change for the better (Antonovsky, 1979).

Some stress theories emphasize the role of appraisal, while others concentrate on the antecedents (Meijman and Mulder, 1998; Geurts and Sonnentag, 2007; Cohen et al., 2016; McEwen et al., 2016). Selye (1956) emphasized the adaptive and subjective nature of stress. Even if the same situation may need different amounts of resources for different people, some situations can be objectively stressful: for example, exceptional and extreme environments.

Extreme environments contain stimuli and/or stressors of such intensity that they can hurt a person's physical and/or psychological well-being (Leach, 2016). 'Exceptional environments' (Leach, 2016) are terrestrial analogs of Space, which are places humans are not naturally prepared for, but can gradually adapt to through coping behaviors (e.g., prisons, polar research stations, etc.). Space and LDSEs are extreme because involving a variety of stressors that would be impossible to deal with without proper training and countermeasures. Indeed, stressors of various types abound in space, originating from a variety of sources (Kanas and Manzey, 2008): physical aspects of the environment, psychological factors of crewmates, habitability variables, and interpersonal dynamics.

One of the most widely used questionnaires in stress research during a space mission is drawn from the sport sciences domain. The Recovery-Stress Questionnaire for Athletes (Rest-Q-Sport; Kellman and Kallus, 2001), which in its latest version has 36 items (Kellman and Kallus, 2016), is a questionnaire that assesses general and specific aspects of stress and recovery. Subjects have to rate the frequency of some behaviors in the previous 3 days/night on a 7-point Likert scale (from ‘never’ to ‘always’). The first half of the questions measure general and specific stress, while the second half general and specific recovery-related feelings and behaviors.

Nicolas and Weiss (2009) evaluated general and specific stress levels among 16 women undergone to a 60-day HDBR. General variables included, for example, anxiety, irritation, social stress, while specific variables included desire to quit, vulnerability to illness, etc. The Rest-Q Sport was used to assess participants at various points before, during, and after their resting period. When they had to shift from a horizontal to a vertical position, both general and specific stress-related responses increased. A recent and ambitious ICC research project that focused on stress and coping responses has been the CELS study (Controlled Ecological Life Support System; Yuan et al., 2019), in which four healthy volunteers (one female) were confined in six interconnected modules for 180 days in order to simulate life on Mars. Even the Martian-like day-night cycle was replicated during the simulation (days 72–108) as one day on Mars lasts approximately 24 h and 40 min. Emotion, stress, and adaptation were measured using behavioral (ethological monitoring), physiological (blood tests), and psychological methods. Throughout the confinement, subjects' stress levels at Rest-Q-Sport remained stable, but scores in fatigue and conflict/pressure subscales tended to increase. The PANAS (Watson et al., 1988) was used to assess emotions, and results showed that, while hostility and negative emotions decreased in intensity during the experiment, positive emotions and anxiety levels remained unchanged, except one male participant, who develop a mild form of depression, reporting increased stress, anxiety, and negative emotions. This general emotions’ trajectory seems to reproduce the one at the Mars500 Project, in which volunteers did not experience an increase in perceived stress or mood disturbances (Basner et al., 2014) but they emotionally adapted, and gave more positive ratings to stimuli with negative valence. According to Wang et al. (2014), this tendency could be the expression of an internal defensive system, which is likely to be triggered by an increase in psychological stress over time. Gemingnani and colleagues (2014) used biological markers to study stress in the 105-day Mars500 Project simulation and discovered that higher cortisol levels were linked to sleep difficulties (for reviews on biological research on stress, see Fogelman and Canli, 2019; Zorn et al., 2017). Similarly, Egawa et al. (2018) measured salivary, skin, and facial image parameters in 23 Japanese subjects who spent two weeks in a confined facility at Tsukuba Space Center. During the confinement period, a link was discovered between stress levels, an increase in salivary cortisol and sebum, and more asymmetrical facial expressions (the distance between the corner of the mouth and the corner of the eyes in the two halves of the face).

Stress in space can come from a variety of sources; additionally, stressors rarely act alone, but rather in concert. Consequently, it may be difficult to detect stress-related mechanisms behind AH disorders. A fruitful way to circumvent this problem is focusing on coping strategies and adaptation skills. Suedfeld's team used Thematic Content Analysis (TCA) to examine coping strategies of active and retired astronauts from their narratives and recounts (Brcic et al., 2018; Suedfeld et al., 2015). Problem-focused strategies (e.g., strategic problem-solving) were the most common used, followed by emotion-oriented strategies (e.g., optimizing one's emotional response); the choice of coping strategies strongly depends on individual differences (e.g., culture, gender, personality traits). Compared to Americans, for instance, Russian cosmonauts use more often active and problem-focused coping strategies rather than emotion-focused ones; male astronauts use more often fight-or-flight strategies (but also more humor-based strategies), whereas female astronauts mostly use tend-and-befriend approach (Taylor et al., 2000). Female orientation, indeed, can help promotion of team cohesion on long-duration missions where crews are heterogeneous (Endler, 2004; Wood et al., 2005), for example, during the EXEMSI simulation, the only female participant among the four members helped to lower tensions (Vaernes, 1993). Brcic et al. (2018) focused in particular humor as one of the most used and powerful emotional coping strategy among astronauts. Authors developed the *Humor Coping Scale,* a scoring guide to assess the frequency and types of humor and humor as a coping strategy, and the followings are two of the items: “Says something amusing in tense situations"; "Laughs in situations where crying or laughing is the only option". Humor, in fact, can be a powerful coping strategy that encourages the cognitive reframing of a stressful situation's affective quality, when functional (i.e. affiliative or self-enhancing), e.g. people with a high sense of humor evaluate situations to be less stressful, and experience less anxiety, than those with a low sense of humor. However, humor can be dysfunctional too (i.e. aggressive or self-destructive) and represent a risk for communications and relationships in a crew during LDSE.

### 2.8 Adaptation and resilience

Humans living and working in ICE environments (e.g., polar stations, offshore platforms, submarines, etc.) are exposed to physical (e.g., harsh climate, light-dark cycle) and psychological stressors (e.g., prolonged isolation, confinement, lack of contacts with their family, limited of privacy, monotony, etc.). Somatic symptoms, sleep difficulties, negative affect (fatigue, depression, anger, and anxiety) impaired cognitive functioning. In addition, Palinkas and Suedfeld (Palinkas, 1991; Palinkas and Suedfeld, 2008) describe interpersonal tension and conflict as part of the winter-over syndrome. Human adaptation is indeed hampered by living in ICE environments. While physical and physiological factors have always been the focus of adaptation research, psychological adaptation processes (PAP) are becoming more popular recently, with the perspective of long-duration and interplanetary missions (Nicolas et al., 2016). Nicolas et al. (2019) developed the ICE-Q (Isolated and Confined Environments Questionnaire) to assess individual and social adaptation in ICE environments. The tool consists of 19 items and considers the four most important adaptation-related domains, according to the literature (Kanas, 2004; Palinkas and Suedfeld, 2008), that is:

- Social domain (5 items), e.g. "Group members support one another");
- Emotional domain (5 items), e.g. "Many of the things I have to do are repetitive and monotonous";
- Occupational domain (4 items), e.g. "I am asked to do too much work";
- Physical domain (5 items), e.g. "I feel physically fit".

Adaptation and stress are interconnected (Selye, 1956). Stress allows physiological and psychological adaptation (Lazarus and Folkman, 1984), but can noxious when excessive or mismanaged (Antonovsky, 1979). In long-duration missions, stress can jeopardize astronauts' well-being, emotional and cognitive health, and psychomotor performance (Kanas and Manzey, 2008; Nicolas et al., 2016). However, stress is not always negative and, when positive (i.e., eustress), can promote adaptation (Kanas and Manzey, 2008). Stress can motivate people and improve learning: moderate levels of stress can make the mission safer by increasing alertness, which, in turn, may increase the chances of survival during an emergency. Furthermore, astronauts often have a positive attitude toward space missions because they gain self-esteem and admiration for space.

In this regard, resilience is one possible mediator between stress and successful adaptation and well-being (Zhang et al., 2021). Resilience can be defined as the ability to stand in the face of negative events or difficulties, and while there is no universally accepted definition of resilience, it is thought to be a significant factor in psychological outcomes (Prince-Embury &, Saklofske, 2014). While some studies consider resilience a personality trait (Bartone, 1999), others a process of social and psychological adaptation (Vanderbilt-Adriance and Shaw, 2008), and still others a subjective response (Edward, 2005), such as rebounding from an adverse social situation, there is debate over the conceptualization and definition of resilience. The ability to actively adapt to adversity and effectively "bounce back" from stressful situations is one of the most widely accepted definitions of resilience (Bonanno, 2004). From a recent meta-analysis, resilience appears to positively relate to well-being and inversely to distress indicators (Hu et al., 2015), and to be a protective factor: resilience would make people less vulnerable to psychosocial distress (Goldstein et al., 2013) and more prone to recover quicker after adversities. Recovery is the dynamic process of the organism returning to a preferred baseline after a state of fatigue (Zijlstra and Sonnentag, 2006); while resilience is an active and dynamic process, recovery is a self-regulation system (Carver and Scheier, 1998).

Zhang and colleagues (2021) studied the relationships between stress, resilience, and adaptation in 103 Chinese Antarctic expeditioners (51 winter-overs and 52 summer-overs; 90% male; M = 39.18, SD = 10.21); the 10.7% of participants were female and all were summer-overs. The 48-item Recovery-Stress Questionnaire (RESTQ-48; Kellman and Kallus, 2001), the ERS, that is, the Essential Resilience Scale (Chen et al., 2016), and the SWLS, that is, the Satisfaction with Life Scale (Diener et al., 1985) were all administered online by the authors. Findings accounted for a negative relationship between resilience and stress, as well as a positive relationship between resilience, recovery, and subjective well-being (SWB). Greater resilience was linked to better stress recovery and subjective well-being. Resilience appears to play a role in increasing happiness, but it is unclear whether positive experiences like professional success can boost resilience, and thus the nature of resilience (e.g., whether it is a personality trait or a learned skill) still needs to be determined. Personal characteristics, as well as resilience, should be considered in space mission planning and in selecting personnel for expeditions in ICE environments. Similarly, Alfano's research group (Alfano et al., 2021; Bower et al., 2019) developed a tool to investigate mental health in extreme environments and future LSDE, the Mental Health Checklist (MHCL), a 23-item self-report questionnaire measured on a 11-point Likert scale ranging from 0 ("never") to 10 ("always") ("always"). Items were created based on interviews with some experts (e.g., NASA psychiatrists, a psychologist with polar experience), as well as a thorough review of the literature. MHCL give scores on three subscales: positive adjustment (e.g., in full control, inspired, determined); poor self-regulation (e.g., restless/fidgety, inattentive, sleepy); anxious apprehension (e.g., worried, obsessed/concerned about things). In a recent study, Alfano et al. (2021) monitored mental health, emotional regulation, physical symptoms, and stress biomarkers in 110 subjects (predominantly white males aged 22–70 years with M = 37.63 and SD = 11.95) in Antarctica during nine months (88 on expedition at McMurdo Coastal Station, and another 22 at South Pole Inland Station). From the beginning to the end of the study, participants showed a significant decrease in positive adaptation and a significant increase in poor self-adaptation at the MHCL. Such decrease in positive adaptation scores over time may indicate detachment and/or indifference; however, increased expression of positive emotions (rather than suppression) and savouring positive experiences appear to slow the decrease in positive adjustment in ICE, which is one of the study's main findings.

In sum, the Antarctic environment shares many stressors with long-duration spaceflight and it is a good analog for studying behavioral changes and AH predictors over time. Moreover, interventions and countermeasures aimed at sustaining and enhancing positive emotions during extended missions (e.g., humor-focused countermeasures) could thus reduce the likelihood of onset of emotional disorders.

### 2.9 Emotions and mood

In the field of space sciences, the study of emotions is crucial. Emotional changes can impair cognitive function, impair mental well-being, and cause psychopathology, especially when it occurs in a stressful situation such as in the deep space for extended periods. Symptoms of depression and anxiety in astronauts have been described as rare: anxiety-related issues occur every 1.2 years, and depressive issues occur every 7.2 years (Slack et al., 2016). Pre-flight selection, psychosomatic manifestations rather than psychological suffering (e.g., asthenia, see Aleksandrovskiy and Novikov, 1996), reluctance to declare psychopathological symptoms for fear of losing flight status, excitement of being in space, and countermeasures, may constitute the reasons. Historically, the lower incidence of mental disorders among astronauts has been attributed to greater emotional stability (Alfano et al., 2017): astronauts would have greater emotional stability than normal people would (e.g., they do not experience negative emotions all of the time, see Mammarella, 2020a), and they would score lower in neuroticism traits (Liu et al., 2016; Landon et al., 2018). The 'right stuff' (Kanas and Manzey, 2008) has been the most widely accepted explanation for astronauts' emotional stability. The personnel selection can be predictive for mission's success in the case of in-Orbit and short-duration missions, but we do not know if it might be the same for out-of-Orbit and interplanetary missions. Due to the multicomponent nature of emotions, studies have often been conducted with different methodologies (e.g., subjective experience, facial expressions, physiological activation, cognitive appraisal, behavioral changes, etc.).

Wang et al. (2014) used a 9-point scale to rate 295 affective stimuli retrieved from the IAPS (International Affective Picture System, see Lang et al., 1997) in terms of valence (positive, negative, and neutral) and arousal during the 520-day Mars500 Project simulation (1 lowest level, 9 highest level). The results revealed a positive bias in participants' ratings, with negative ratings decreasing over time. According to the Process Model of emotion regulation (Gross, 2015, Gross et al., 2019), this can be due to either a low emotional response to unwanted stimuli or attentional deployment, i.e., the explanation for such phenomena can be found at two different points in the emotion regulation process. Solcova and colleagues (2014) used the Mood Adjective Checklist (UWIST) (Matthews et al., 1990) and a checklist of emotion frequency and regulation to assess mood, emotions, and emotional regulation during the Mars520 project. Participants were asked to rate their mood in the previous week using an adjective on a 5-point scale (from 1 to 5). There were 12 adjectives total: six positive (happy, cheerful, competitive, optimistic, satisfied, happy) and six negative (low mood, unsatisfied, gloomy, depressed, sad, and sorry). Then they rated how often they had experienced a series of emotions (such as joy, sadness, fear, optimism, embarrassment, and so on) and how much they had hidden them from others on a 4-point scale (that is, which strategies they used to regulate their emotions). The crew reported a higher number of adjectives associated with a positive mood and positive emotions, indicating a consistent predominance of positive emotions throughout the mission (except a few isolated cases); in general, they immediately expressed their positive emotions to crewmates while attempting to avoid negative ones. The analysis of mood correlations and fluctuations revealed that, while the crew's affective tone was predominantly positive, the specific moods were not experienced synchronously, and that when one crewmember's mood increased, it was compensated by a decrease in the mood of the other. In light of the social identity theory (Stets and Burke, 2000; Tajfel et al., 1979) and the group-based emotion generation/regulations theory (Mackie et al., 2000; Goldenberger et al., 2016; Smith, 1993), this last result appears to be intriguing. When someone categorizes himself as a member of a group (e.g., an association), he may begin to participate in that group's emotional life, i.e., he becomes an active regulator of the group's emotions, just as he does with his own subjective emotions. Another LSDE-related emotional phenomenon is displacement (or detachment, see Kanas and Manzey, 2008), which involves shifting tensions externally. During simulations, the research team became nervous and projected their aggression and dissatisfaction outward, blaming the on-ground mission control personnel (MCC), who were the furthest away and least able to react. Detachment has been observed during both analog simulations, such as the Mars520 (Jackson et al., 1972; McDonnell Douglas Astronautics Company, 1968), SIRIUS-17, SIRIUS-19, in which communications between the crew and MCC gradually decreased (Supolkina et al., 2021), and real missions (Lebedev, 1988). Detachment could be a coping or emotion regulation strategy used by a group. Future research into this phenomenon and its relationship to positive and negative affect is required.

In order to assess the effect of 45 days of HDBR on emotions and executive function in 16 healthy young men at six different time points, Liu et al. (2012) used an emotional flanker task (pre-HDBR, HDBR-11, HDBR-20, HDBR-32, HDBR-40, post-HDBR). They first evaluated participants’ affects at the PANAS, finding a significant main effect of positive affect at various time points. The mean positive affect decreased dramatically at the start (HDBR11), then increased in HDBR20, and then gradually decreased in subsequent measurements and post-HDBR (with a significant difference from pre-HDBR). They found no significant differences in levels of negative affect, anxiety, or depression, in contrast with a previous study of Ishizaki et al. (2002), who found levels of depression in nine young men to increase during a 20-day HDBR period. Then, for the emotional flanker task, subjects had to press a specific button to identify the emotion of a target face between three presented. There could be two conditions: congruent, i.e. three faces all expressed the same emotion, vs. incongruent. Accuracy and reaction time were better in the congruent condition, as one might expect. Not accuracy but reaction times significantly worsened from pre-HDBR to any other measurement, including post-HDBR. These findings suggest that prolonged bed rest can deteriorate executive functioning and positive affect, but whether this is due to the lack of aerobic physical activity or the effect of HDBR itself is to establish.

Another study (Brauns et al., 2019) in this field used event-related potentials to investigate the effect of a 30-day session of a -6°-tilted-HDBR on cortical emotional modulation. Participants were twenty young men, who were divided into two groups (HDBR vs. controls), and rated 75 IAPS affective stimuli in terms of valence and arousal. Compared to controls, HDBR subjects had significantly lower P300 and LPP amplitudes in the centroparietal regions (particularly in the posterior cingulate gyrus, insula, and precuneus) in response to pleasant and unpleasant, but not neutral, emotional stimuli. This could mean that HDBR subjects have less cognitive resources both in attention and memory storage (as indicated by the P300) and in encoding and memory processes (as indicated by the LPP) when they look at an emotional image. In a separate study (Mélendez et al., 2020), 84 subjects significantly worsened in an emotion recognition task from before to during Covid-19 lockdown, compared to controls (n = 80). In particular, the confined group had a decrease in recognizing expressions of happiness and got worth in recognizing sadness at the Ekman 60 Faces Test (Young et al., 2002). Interestingly, no significant results were found in relation to anger, fear, disgust, or surprise recognition. If we assume that lockdown isolation is comparable to isolation/confinement in ICE environments, this study could be relevant to space scientists. It would be beneficial to replicate this kind of research during HDBR period in order to assess emotion recognition in microgravity-like conditions.

Emotion research in the space sciences is needed not only to manage affective health, but also to address the effects of emotions on astronauts' cognitive function, behavior, and performance. Emotions, for example, can influence – and even distort – time perception, which is critical to spaceflight success (the 'time-emotion paradox,' see Droit-Volet et al., 2009). Qian and colleagues (2021) investigated the effects of emotional stimuli on time perception and changes in 16 subjects during a 15-day HDBR period in microgravity-like conditions (over 7 days of pre-HDBR and another 7 days of post-HDBR, for a total of 29 days). They used a temporal bisection task to assess time estimation when emotional stimuli were presented. Volunteers memorized two standard durations, one short (300 ms) and one long (900 ms), in the first phase (learning phase). Then, in the second phase, they were asked to press "A" if the image presentation time was closer to the short standard or "L" if it was closer to the long standard. The experimental session was divided into eight blocks, each one containing 21 trials. Every image could elicit fear, disgust, or no emotion (neutral); images were shown randomly and for different duration of time (300, 400, 500, 600, 700, 800, 900 ms). Finally, volunteers rated every images on the 9-point Manikin Self-Assessment Scale (Bradley and Lang, 1994) in arousal and valence. The experiment was conducted at different time points: on the third day of the pre-BR period, the eighth (BR-Mid), the fifteenth (BR-Late) day of the HDBR period, and the fifth day of the post-BR period. The task was performed in supine position during the HDBR period vs. sitting position in pre- and post-HDBR phases. Results revealed two concurrent trends: first, a temporal overestimation for fear stimuli in the BR-mid (day 8), BR-late (day 15), and post-BR phases, compared to the pre-BR phase, and, second, the general decrease in temporal sensitivity in the middle and later phases, particularly for fear and neutral stimuli. During the HDBR, participants made significantly – and progressively – more errors in estimating the presentation time of fearful and neutral stimuli compared to disgust-related stimuli. These results could be influenced by several factors, including stimuli selected, head-down position, and physiological changes in bed rest participants, such as reduced vagal excitability. It is possible, for example, that subjects become less aware of their body state as HDBR progresses (similar to the reduction in proprioceptive feedback in astronauts in a microgravity space environment, see Semjen et al., 1998). To avoid errors in spatial operations, the impact of the microgravity environment and the accompanying changes in psychological and physiological factors on time perception should be considered.

### 2.10 SOC orientation and positive effects of being in space

As stated before, space missions can be stressful, but they can also bring positive aspects that help astronauts grow personally, e.g., many astronauts returned to Earth with a more optimistic outlook on themselves and humanity (Connors et al., 1985; Kanas and Manzey, 2008). Being in space is reported to be a massive experience with a long-lasting impact on astronauts' AH (Palinkas, 1991; Suedfeld, 1998; Suedfeld et al., 2010). At the same time, the success of a space mission depends on one's ability to maintain a positive attitude: the positive arousal associated with their mission appears to be one of the strongest factors that improved communication with crewmates and ground control personnel (Kelly and Kanas, 1992, 1993). The perception of the Earth can be a critical factor in maintaining a positive mindset (Suedfeld et al., 2012), e.g., Lebedev (1988) wrote in his diary that a photograph of the Earth helped him to cope with his emotional problems during the mission. Ihle, Ritsher, and Kanas (2006) developed the Positive Effects of Being in Space (PEBS) questionnaire and administered it anonymously to 175 astronauts and cosmonauts who had flown on at least one mission. They found that every respondent had a positive reaction to being in space such as being awe at the Earth's beauty and fragility. An explanation of this can be found in the salutogenic approach (Antonovsky, 1979), which considers health and happiness depending on the subjective sense of coherence (SOC). The SOC is the belief that stimuli (internal and external) are meaningful, predictable, and manageable. Finding a meaningful coherence in own life and experiences has been found to correlate with good health perception (Eriksson and Lindström, 2006; Honkinen et al., 2005) and with fewer psychological symptoms (Blom et al., 2010; Ristakari et al., 2008).

More recently, Suedfeld et al. (2012, 2015) compared positive changes in personal growth between 20 retired male astronauts and two groups of people on Earth who had experienced stressful events (mothers for the first time vs. trauma survivors). The astronauts' group scored higher in two areas, i.e., realizing new possibilities and personal strength. They were also assessed for coping strategies: astronauts used more problem-focused coping strategies rather than emotion-oriented ones compared to the Earth group. The SOC orientation may be a key feature of astronauts' emotional stability and keeping in mind the mission's superordinate goals may explain why spaceflights are beneficial and act as a mediator for emotional health and well-being. We agree with Suedfeld (2001, 2005) about the prioritization of positive psychology and salutogenesis in planning future spaceflights.

### 2.11 Which countermeasures for long-duration space missions?

Kanas (2015) distinguishes between countermeasures aimed to ameliorate ergonomic aspects of the spacecraft (e.g., environmental engineering) and those aimed to improve skills and habits to favor adaptation and well-being (e.g., scheduling work and rest, see Fitts, 2000). Both aspects are important in an interplanetary mission. Indeed, for basic life support and operational activities, astronauts on their way to Mars will rely heavily on computers and other devices on board, so improving the ergonomics of the human-machine interface is a priority. Similarly, it will be necessary to balance crewmate interactions and personal leisure time.

Kanas (2015), moreover, considered three domains of countermeasures, that is, pre-flight preparation, in-flight support, and post-mission readaptation. Crewmember selection and pre-flight training are the most used pre-flight countermeasures and include seminars, field exercises, and team-building exercises for providing astronauts with psychological knowledge and skills (e.g., coping with environmental stressors and communicating with crewmates and ground personnel). In-flight countermeasures are directed to monitor and support crewmembers' well-being and can be provided both on-board and remotely, e.g., via periodic self-reports assessing emotional and cognitive states, audio-visual observations, and behavioral analysis. In this regard, voice stress analysis is a promising tool under development (Lieberman et al., 2005). Nevertheless, onboard monitoring can be necessary for LSDEs and could be done by crewmembers themselves, who may be trained to detect potential problems among their crewmates and manage or alert crew or ground personnel. Astronauts, for instance, could be given formal self-monitoring instruments, similarly to what already happens for evaluating cognitive (Shephard and Kosslyn, 2005a, Shephard and Kosslyn, 2005b) and neurocognitive functions (Retzlaff et al., 1999). In LDSEs, on-board support activities will be critical and will be strongly diverse from the on-orbit measures used so far (e.g., surprise gifts and foods delivered, increased contact with people on Earth, and psychological support given by ground-personnel, see Kanas et al., 1996). Of course, some of these support tools can be used in long-duration missions, while others will need to be adjusted or replaced with others more functional and useful. Onboard music and lighting, for example, are commonly used during short-duration spaceflights and can be maintained even during long-duration ones. Computerized self-help tools and micro-courses on psychosocial education, as well as group sessions to share personal experiences and discuss potential stressors before they become problematic, would all be beneficial. A voice-to-text transcription device may facilitate communication between onboard members and ground personnel (Love and Reagan, 2013). The "Disappearing Earth" phenomenon is the progressive rise in homesickness and melancholy associated with increasing distance from the home planet. This will be a fact of special interest for LSDEs if we consider that during the Mars-500 long-term isolation study, the prolonged lack of visual contact with Earth has been associated with a decrease in crew activity and motivation, increasing independence from the ground-personnel recommendations, aggression, and "groupthink" (Gushin et al., 2021; Kanas et al., 2009). Kanas (2015) proposes developing an onboard telescope that allows crewmembers to see the Earth in real-time, which could help them cope with feelings of separation; Gushin et al. (2021) believe that with volumetric video or 3D computer models, virtual reality (VR) may be the preferred tool for presenting Earth's nature and everyday life images to astronauts. VR can also be used to organize forms of leisure to fight boredom, as well as to create a virtual personal space to compensate for the lack of physical personal space; crewmembers would be able to create their own virtual homes. A greenhouse on board could be a promising countermeasure, as it can help astronauts feel more connected to the Earth while also providing a food source that adds variety to the astronauts' diet. Greenhouses on board are also effective for psychological relaxation, as they have a positive effect on crews' emotional states in both spaceflight and ICE isolation sessions; in addition, in their leisure time, astronauts could care plants and break the monotony of the flight (Gushin et al., 2021).

Finally, after the mission is completed and the astronaut and his or her family return to Earth, readjustment issues can arise, especially after a long-duration space mission. As a result, post-mission readaptation measures such as joint debriefings of crewmembers and their families by trained consultants must be developed (Kanas, 2015).

### 2.12 Psychological countermeasures and Affective Health Training in LSDEs

Psychological countermeasures are a set of actions aimed at reducing the negative effects of spaceflight's extreme living and working conditions on crewmembers' cognitive performance and well-being (Kanas and Manzey, 2008). To date, psychological research in the space sciences has focused almost exclusively on anxiety and stress management training. Stress Exposure Training (Driskell and Johnson, 1998; Cuevas, 2003), for example, involves a first training phase (on knowing typical stress reactions and coping skills) and, a second application phase, in which future astronauts practice what they have learned in various proposed scenarios. Blue et al. (2017) developed a training program for spaceflight-related stress in order to mitigate astronauts' anxiety before and during a launch simulation. Jing et al. (2011), similarly, used guided imaging to reduce stress during centrifuge training, with significant results compared to control subjects who simply listened to relaxing music. In long-duration missions, however, stress will not be the only issue to manage, and, over the past 10 years, interest in emotional training has grown significantly. Emotional trainings in general aim to increase emotional regulation and perceived control skills (Kanas, 2015; Mammarella, 2020a, 2020c; Schweizer et al., 2011, 2013), but an Affective Training (AT), or an Affective Health Training (AHT) should include countermeasures for both emotions, stress, and mood. Guided imagination, which is useful in improving astronauts' performance during emergency tasks (Yijing et al., 2015), could easily be included in emotional training for LSDEs. Mindfulness and relaxation techniques could also be valuable assets for long-duration missions (Pagnini et al., 2019). Indeed, mindfulness practice positively correlates with several important characteristics for long-term missions, such as emotional stability, ability to enjoy solitude, empathy and sensitivity, low need for order (Palinkas et al., 2002), social adaptive skills (Palmai, 1963), high tolerance for boredom (Lugg, 2005). Mindfulness-based interventions and relaxation training, including cognitive reorganization skills and breath control exercises, can be effective in mitigating negative consequences of lack of sleep, isolation, and boredom, can improve concentration and attention, with important implications for emergent tasks and extravehicular activities. In this regard, voice assistants based on artificial intelligence of social robotics (Kemke, 2007), which could provide psychological assistance to astronauts who are unable to communicate directly with family and friends due to communication delays, can play a major role. These kinds of devices are currently used in some medical and psychological fields, such as psychological rehabilitation, and could also aid astronauts in both technical and everyday tasks, such as leisure organization (presentation of news, references, and entertainment information, interactive games with voice support), and psychotherapy based on active listening. In addition, an AHT for long-duration space exploration may include VR technology, which has already been tested by NASA for psychological support in extreme environments (Anderson et al., 2016), and also in recent dry immersion session, with satisfactory results in both emotional and cognitive domains (Rozanov et al., 2021; Nosenkova, 2021). An AHT should finally involve a training program to reinforce the personal meaning associated with the mission (Britt et al., 2017), the sense of coherence (Antonovsky, 1979), and humor (Brcic et al., 2018). Humor is an extremely effective coping strategy and could be leveraged in LSDEs to both increase positive emotions and regulate crew mood in times of generalized nostalgia or low mood. Finally, another valuable tool for an affective training for LSDEs can be the one developed from Botella and colleagues (2016) for the Mars500: the Emotional Activities Related to Health (EARTH) program. EARTH is a computer program aimed at systematically improving the well-being and mood of astronauts with three applications to which users can access whenever they want and that point to recall positive memories and autobiographical events related to life on Earth.

## 3 Discussion

Psychiatry has been interested in human adaptation since the early days of space exploration. One of the first to propose a three-phase model of emotional adaptation to space was Rohrer (1961). Following a first phase characterized by a high level of anxiety (due to the perceived danger and novelty), a second phase would show, in which astronauts may experience depressive symptoms due to the temporary loss of their usual social roles (husband, wife, son/daughter, etc.). In the third and final phase, astronauts adapt to the space experience and express their emotions and some irritability. Gushin et al. (1993) revised this model, proposing four phases going from an initial discomfort due to physical symptoms (e.g., motion sickness), to an emotional balance, to a phase of emotional instability (in which astronauts experience irritability and lower energy levels), to a euphoric mood. Both Rohrer's and Gushin's models revealed physiological emotional instability in astronauts' experience during a space mission, even though they are typically considered emotionally stable. That dramatic phase, according to Bechtel and Berning (1991), would not be dependent on the mission's duration but would occur three-quarters of the way through the mission. Astronauts would be more likely to develop emotional disturbances and interpersonal problems during this period of high emotional instability. This phenomenon could be explained by the fact that astronauts, regardless of the mission duration, tend to segment their missions in three periods (beginning, middle, and end). When half of the mission is completed, they realize that an equally long period of isolation awaits them and, as a result, their negative emotions tend to rise. Although this phenomenon has only been observed in analogs, and empirical evidence for its existence on the ISS is scarce (Kanas and Manzey, 2008), it attests to the effort and insights of early psychiatrists and psychologists who pondered about Space challenges for humans, which imply the full consideration and management of human faculties and resources. Indeed, interest in astronauts' emotions has progressively increased since the description of the three-quarter phenomenon (Alfano et al., 2017), especially in the light of long-duration missions. We know how important positive affects are for achieving goals and for broadening not only our thoughts but also our action possibilities (Fredrickson, 2004; Fredrickson et al., 2008). We also know that focusing solely on emotions and ignoring other health components, such as resilience, may not be enough to keep astronauts healthy for months in space. In this review, we argument that emotions, coping strategies, resilience, and personal constructs related to a space mission, in addition to stress and mood (which are undoubtedly the most investigated constructs in space sciences psychological research), are all equally important to adaptation and, ultimately, to the affective health during a LDSE. Due to a lack of research on the topic and the assumption that AH is a complex construct, confusions may arise. However, in light of Gross's (2015) model, we wanted to emphasize that emotions are only one type of human affect, and that the definition of AH necessitates consideration of multiple emotion components. Strictly, we assumed that, in space sciences, it would be more accurate to refer to AH as the proportion of health resulting from efficient affective regulation processes, such as mood, stress, coping. We believe that, besides its inclusiveness, the concept of AH can be a theoretical driver for studying and monitoring the health of astronauts on long-duration missions. We hope that future research in this area will be able to systematize the concept better and develop a validated theoretical model of AH in space missions, so that we can better understand how affects and cognitive functions interact, as well to promote successful adaptation to extreme environments. For example, although astronauts' coping strategies appear to indicate the use of cognitive and controlled processes to prevent the occurrence of negative emotions, we are unaware of any research on emotion regulation processes in terms of bottom-up (e.g., inhibition) and top-down (e.g., cognitive reappraisal). Moreover, the field of adaptation processes to Space and long-term missions requires a diversity of approaches, which includes not only interdisciplinary but also simultaneous attention to group dynamics. We will be able to conceptualize, promote, and protect AH in long-term missions only if we gain a better understanding of the cognitive and affective aspects, as well as their intrapsychic and interpersonal interactions among crewmembers.

## Declarations

### Author contribution statement

Matteo Gatti; Rocco Palumbo; Alberto Di Domenico; Nicola Mammarella: All authors listed have significantly contributed to the development and the writing of this article.

### Funding statement

This research did not receive any specific grant from funding agencies in the public, commercial, or not-for-profit sectors.

### Data availability statement

No data was used for the research described in the article.

### Declaration of interests statement

The authors declare no conflict of interest.

### Additional information

No additional information is available for this paper.
